# Does high-grade dysplasia/carcinoma in situ of the biliary duct margin affect the prognosis of extrahepatic cholangiocarcinoma? A meta-analysis

**DOI:** 10.1186/s12957-019-1749-7

**Published:** 2019-12-09

**Authors:** Qiao Ke, Bin Wang, Nanping Lin, Lei Wang, Jingfeng Liu

**Affiliations:** 1 Department of Hepatopancreatobiliary Surgery, Mengchao Hepatobiliary Hospital of Fujian Medical University, Xihong Road 312, Fuzhou, 350025 Fujian China; 2 Department of Pathology, School of Basic Medical Sciences of Fujian Medical University, Fuzhou, China; 3 Department of Pathology, Mengchao Hepatobiliary Hospital of Fujian Medical University, Fuzhou, China; 4 Department of Radiation Oncology, Mengchao Hepatobiliary Hospital of Fujian Medical University, Fuzhou, China; 5 Liver Disease Center, The First Affiliated Hospital of Fujian Medical University, Fuzhou, China

**Keywords:** Extrahepatic cholangiocarcinoma, High-grade dysplasia, Carcinoma in situ, Prognosis, Meta-analysis

## Abstract

**Background:**

High-grade dysplasia/carcinoma in situ (HGD/CIS) of the biliary duct margin was found to not affect the prognosis of patients with extrahepatic cholangiocarcinoma by recent studies, but it has not yet reached a conclusion.

**Methods:**

Eligible studies were searched by PubMed, PMC, MedLine, Embase, the Cochrane Library, and Web of Science, from Jan. 1, 2000 to Jun. 30, 2019, investigating the influences of surgical margin status of biliary duct on the prognosis of patients with resectable extrahepatic cholangiocarcinoma. Overall survival (OS) and local recurrence were evaluated by odds ratio (OR) with 95% confidence interval (CI).

**Results:**

A total of 11 studies were enrolled in this meta-analysis, including 1734 patients in the R0 group, 194 patients in the HGD/CIS group, and 229 patients in the invasive carcinoma (INV) group. The pooled OR for the 1-, 2-, and 3-year OS rate between HGD/CIS group and R0 group was 0.98 (95% CI 0.65~1.50), 1.01 (95% CI 0.73~1.41), and 0.98 (95% CI 0.72~1.34), respectively. The pooled OR for the 1-, 2-, and 3-year OS rate between HGD/CIS group and INV group was 1.83 (95% CI 1.09~3.06), 4.52 (95% CI 2.20~9.26), and 3.74 (95% CI 2.34~5.96), respectively. Subgroup analysis of extrahepatic cholangiocarcinoma at early stage showed that the pooled OR for the 1-, 2-, and 3-year OS rate between HGD/CIS group and R0 group was 0.54 (95% CI 0.21~1.36), 0.75 (95% CI 0.35~1.58), and 0.74 (95% CI 0.40~1.37), respectively, and the pooled OR for the 1-, 2-, and 3-year OS rate between HGD/CIS group and INV group was 3.47 (95% CI 1.09~11.02), 9.12 (95% CI 2.98~27.93), and 9.17 (95% CI 2.95~28.55), respectively. However, the pooled OR for the incidence of local recurrence between HGD/CIS group and R0 group was 3.54 (95% CI 1.66~7.53), and the pooled OR for the incidence of local recurrence between HGD/CIS group and INV group was 0.93 (95% CI 0.50~1.74).

**Conclusion:**

With the current data, we concluded that HGD/CIS would increase the risk of local recurrence compared with R0, although it did not affect the prognosis of patients with extrahepatic cholangiocarcinoma regardless of TNM stage. However, the conclusion needs to be furtherly confirmed.

## Introduction

The incidence of extrahepatic cholangiocarcinoma including hilar and distal cholangiocarcinoma is increasing stably [1, 2], but the prognosis is generally poor [3, 4]. Surgical resection is still the only potential way to achieve a long survival [4–6], although most of the patients have lost the chances of surgery at diagnosis [7, 8]. However, the 5-year survival rate remains far away from satisfactory even if surgery has been undergone [5, 6], partly because radical resection is hard to achieve for extrahepatic cholangiocarcinoma in anatomy [5, 6].

Margin status is deemed to be associated with prognosis of extrahepatic cholangiocarcinoma [9–11]. Additional resection and adjuvant treatments are often necessary if surgical margin is positive [12–14], although there is no consensus after R0 resection to deliver adjuvant treatments for resected bile duct cancer [15]. But, several newly published studies showed that compared with invasive carcinoma (INV), high-grade dysplasia/carcinoma in situ (HGD/CIS) of the biliary duct margin did not affect the prognosis of patients with operable cholangiocarcinoma, and additional resection did not improve the prognosis when the margin was HGD/CIS [12, 16–18]. However, most of the studies were single-center, and the sample size was small. Hence, a meta-analysis was warranted to confirm whether HGD/CIS could affect the prognosis of patients with resectable extrahepatic cholangiocarcinoma.

## Material and method

This study was based on the published reports; hence, the informed consent of the patients and the ethical approval were not required. This meta-analysis was conducted according to the Preferred Reporting Items for Systematic Reviews and Meta-Analyses (PRISMA).

### Literature search

A comprehensive search on the existing published medical literature was conducted by Qiao Ke and Nanping Lin to investigate the influences of surgical margin status of biliary duct on the prognosis of patients with resectable extrahepatic cholangiocarcinoma. English electronic databases such as PubMed, PMC, MedLine, Embase, the Cochrane Library, and Web of Science were used to search the literature from Jan. 1, 2000 to Jun. 30, 2019. Keywords were as follows: (“cholangiocarcinoma” or “extrahepatic cholangiocarcinoma” or “bile duct cancer” or “bile duct carcinoma” or “hilar cholangiocarcinoma” or “perihilar cholangiocarcinoma” or “HCCA” or “PHC” or “Klatskin’s tumor” or “distal cholangiocarcinoma”) AND (“margin” or “duct margin”). Any potentially eligible studies were then identified manually through the references of the included studies, reviews, letters, and comments.

### Selection criteria

Inclusion criteria are as follows: (i) patients with either hilar or distal cholangiocarcinoma; (ii) margin status was confirmed by either intraoperative frozen pathology or postoperative pathology; (iii) groups must include HGD/CIS group, in which the margin status was either HGD or CIS; (iv) outcomes must include overall survival (OS) rate.

Exclusion criteria are as follows: (i) patients including gallbladder carcinoma; (ii) data on the OS rates was not available; (iii) studies based on overlapping cohorts deriving from the same center.

### Intervention

Major hepatectomy or caudate lobectomy with extrahepatic bile duct resection was generally for hilar cholangiocarcinoma [8, 17], and the standard Whipple’s procedure for distal cholangiocarcinoma [12]. Of note, regional lymph nodes were dissected in all procedures.

Both distal margin and proximal margin were collected to conduct an intraoperative frozen section examination and were evaluated by at least two pathologists within 30 min [8]. Additional resection was performed if possible when either distal margin or proximal margin was positive, but it mainly depended on each center [12, 18, 19].

Specially, it was extremely difficult to distinguish between high-grade dysplasia (HGD) and carcinoma in situ (CIS) [20]. Herein, in this study, we classified them into HGD/CIS group.

### Endpoints

Primary endpoints were 1-, 2-, and 3-year survival rates either between HGD/CIS group and R0 group or between HGD/CIS group and INV group. Secondary endpoints were the incidence of local recurrence either between HGD/CIS group and R0 group or between HGD/CIS group and INV group.

### Data extraction

Data such as the author’s first name, year of publication, study methods, patient’s characteristic, interventions, and outcomes were extracted and assessed by Qiao Ke and Nanping Lin according to the predefined forms. The odd ratios (ORs) of 1-, 2-, and 3-year survival rates were extracted directly from the original data or extracted from the Kaplan-Meier curves according to the methods described in detail by Tierney et al. [21] and Parmar et al. [22]. In case of disagreement, a third investigator, Bin Wang, was intervened to reach a conclusion.

### Quality assessment

The quality of non-randomized studies was assessed by the modified Newcastle-Ottawa Scale (NOS) [23], and more than 7 stars were defined as high quality, 4~6 star as medium quality, and < 4 stars as low quality.

### Statistical analysis

The meta-analysis was registered at http://www.crd.york.ac.uk/PROSPERO/ (Review registry 142411) and was performed using RevMan Version 5.3. The outcomes between HGD/CIS and R0 or invasive carcinoma were evaluated by ORs and 95% CIs. To choose whether random effects or fixed effects mode, the heterogeneity was assessed by the *χ*^2^ test and *I*^2^ statistics; *P* < 0.10 or *I*^2^ > 50% were considered as significant heterogeneity. When the hypothesis of homogeneity was rejected, the fixed effects model was used to estimate the case with homogeneity, and the random effects model was used for the cases with significant heterogeneity [24, 25]. Sensitivity analysis was conducted as follows: one study at a time was removed and the remained were re-analyzed to determine whether the results could be affected significantly by single study [26]. Begg’s and Egger’s tests were used to evaluate publication bias using Stata 14, and “trim and fill” method was conducted to test the influence of publication bias on the overall outcomes if the funnel plots were found asymmetric [26]. *P* < 0.05 was considered statistically significant, and all *P* values were two-tailed.

## Results

### Base characteristic of the included studies

**Fig. 1:**
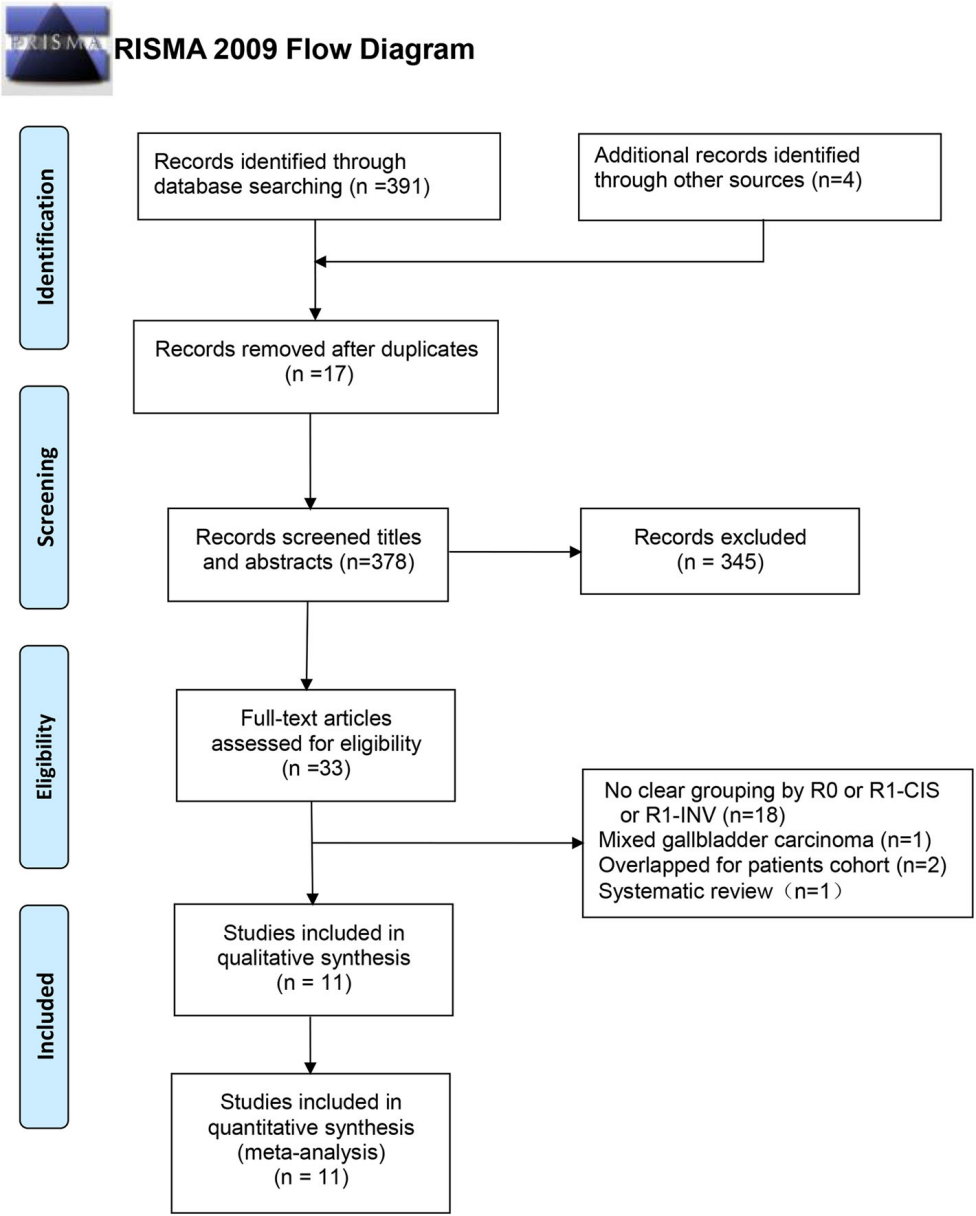
PRISMA flow diagram showing selection of articles for meta-analysis

A total of 395 records were identified by Qiao Ke and Nanping Lin, including 391 records through electronic search and four records through manual search. Seventeen records were excluded for duplication by NoteExpress 3.1, and then 345 records were excluded after browsing titles and abstracts. Finally, 22 records were excluded after full-text review for the following reasons: (i) 18 records for unclear grouping; (ii) two records for overlapped cohorts [27, 28]; (iii) one record for mixed gallbladder carcinoma [29]; (iv) one record for review [16]. Hence, 11 records were enrolled into our meta-analysis [12, 17–19, 30–36]. The search strategies and results are shown in Fig. 1.

Totally, 2157 patients including 1734 patients in the R0 group, 194 patients in the HGD/CIS group, and 229 patients in the invasive carcinoma (INV) group were included into this study. The characteristics and baseline demographic data of the patients in each research are listed in Table 1. Of note, almost all of the studies came from Japan and South Korea [12, 17–19, 30, 32–36], and only one came from the USA [31]. The incidences of HGD/CIS and INV ranged from 3.0 to 19.5% and 3.5 to 18.3%, respectively. A total of 10 studies were scored above 6 by NOS [12, 17–19, 30, 31, 33–36], and only one study was scored 6 [32].

**Table 1:** Characteristics of the clinical trials included in the meta-analysis

Study	Country	Study years	Sex (F/M)	Lymphatic invasion (yes/no)	Perineural invasion (yes/no)	Differentiation (well/moderate poor)	Median follow-up (months)	Location	Ductal resection margin status	Patients	Quality
Sasakai, 2007 [30]	Japan	1985–2005	NR	115/13	102/26	50/78	NR	Perihilar (*n* = 51) Distal (*n* = 77)	R0	105	7
									R1-CIS	12	
									R1-INV	11	
Endo, 2008 [31]	USA	1992–2005	NR	NR	NR	NR	26 ± 2.5(1–103)	Perihilar (*n* = 82)	R0	66	7
									R1-CIS	16	
Igami 2009 [32]	Japan	1977–2005	162/309	NR	NR	NR	NR	Perihilar (*n* = 351) Distal (*n* = 120)	R0	410	6
									R1-CIS	14	
									R1-INV	47	
Nakanishi, 2010 [33]	Japan	1989–2007	25/100	NR	106/19	38/87	NR	Perihilar (*n* = 103) Distal (*n* = 22)	R0	96	8
									R1-CIS	10	
									R1-INV	19	
Wakai, 2011 [34]	Japan	1988–2007	41/69	NR	NR	27/83	99 (1–259)	Perihilar (*n* = 57) Distal (*n* = 53)	R0	85	8
									R1-CIS	14	
									R1-INV	11	
Han, 2013 [35]	South Korea	1995–2007	129/335	NR	NR	NR	32 (1–181)	Perihilar (*n* = 208) Distal (*n* = 246) Diffuse (*n* = 10)	R0	340	8
									R1-CIS	39	
									R1-INV	85	
Oguro, 2015 [36]	Japan	2000–2011	49/100	NR	NR	42/107	26.5 (2.20–143.7)	Perihilar (*n* = 149)	R0	128	7
									R1-CIS	21	
Kurahara, 2016 [18]	Japan	2002–2014	27/73	NR	NR	49/51	25.3	Perihilar (*n* = 35) Distal (*n* = 65)	R0	69	8
									R1-CIS	16	
									R1-INV	15	
Tsukahara, 2017 [19]	Japan	1998–2013	59/113	66/106	103/69	93/79	86.9	Perihilar (*n* = 144) Distal (*n* = 28)	R0	148	8
									R1-CIS	18	
									R1-INV	6	
Higuchi, 2017 [17]	Japan	2004–2013	58/105	NR	NR	NR	NR	Perihilar (*n* = 163)	R0	113	7
									R1-HGD/CIS	22	
									R1-INV	28	
Park, 2019 [12]	South Korea	2008–2016	83/110	NR	137/56	42/151	35.9 (22.4–58.4)	Distal (*n* = 193)	R0	174	7
									R1-CIS/HGD	12	
									R1-INV	7	

### Primary endpoint

**Fig. 2:**
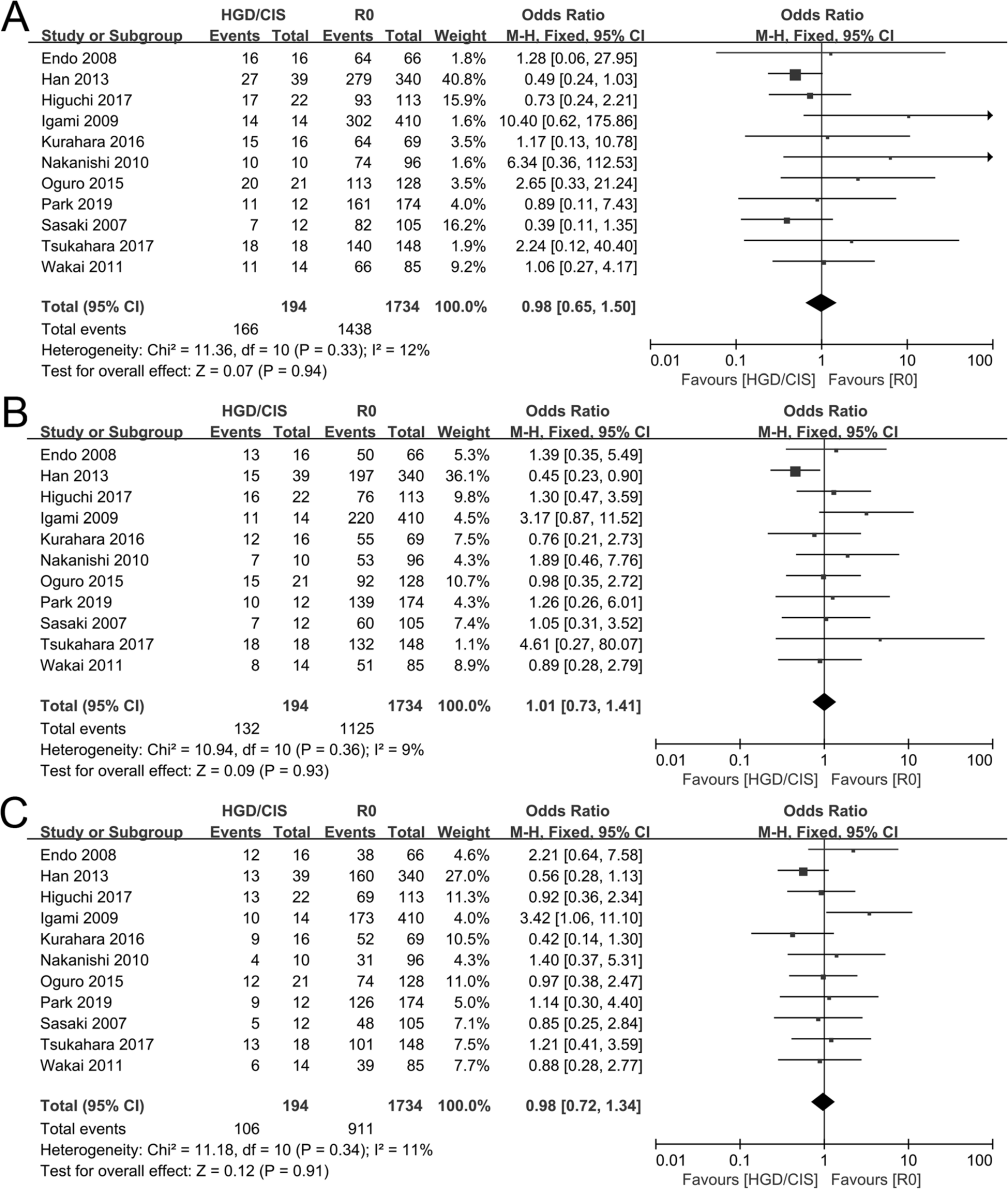
Forest plot of overall survival rates between HGD/CIS group and R0 group. **a** 1-year overall survival rate. **b** 2-year overall survival rate. **c** 3-year overall survival rate

The 1-, 2-, and 3-year survival rates comparing between HGD/CIS group and R0 group were evaluated in 11 included studies [12, 17–19, 30–36]. Significant heterogeneities were not observed (*I*^2^ = 12%, *P* = 0.33; *I*^2^ = 9%, *P* = 0.36; *I*^2^ = 11%, *P* = 0.34; respectively), and using a fixed model, the pooled OR for the 1-, 2-, and 3-year survival rate between HGD/CIS group and R0 group was 0.98 (95% CI 0.65~1.50, *P* = 0.94, Fig. 2a), 1.01 (95% CI 0.73~1.41, *P* = 0.793, Fig. 2b), and 0.98 (95% CI 0.72~1.34, *P* = 0.91, Fig. 2c), respectively.

**Fig. 3:**
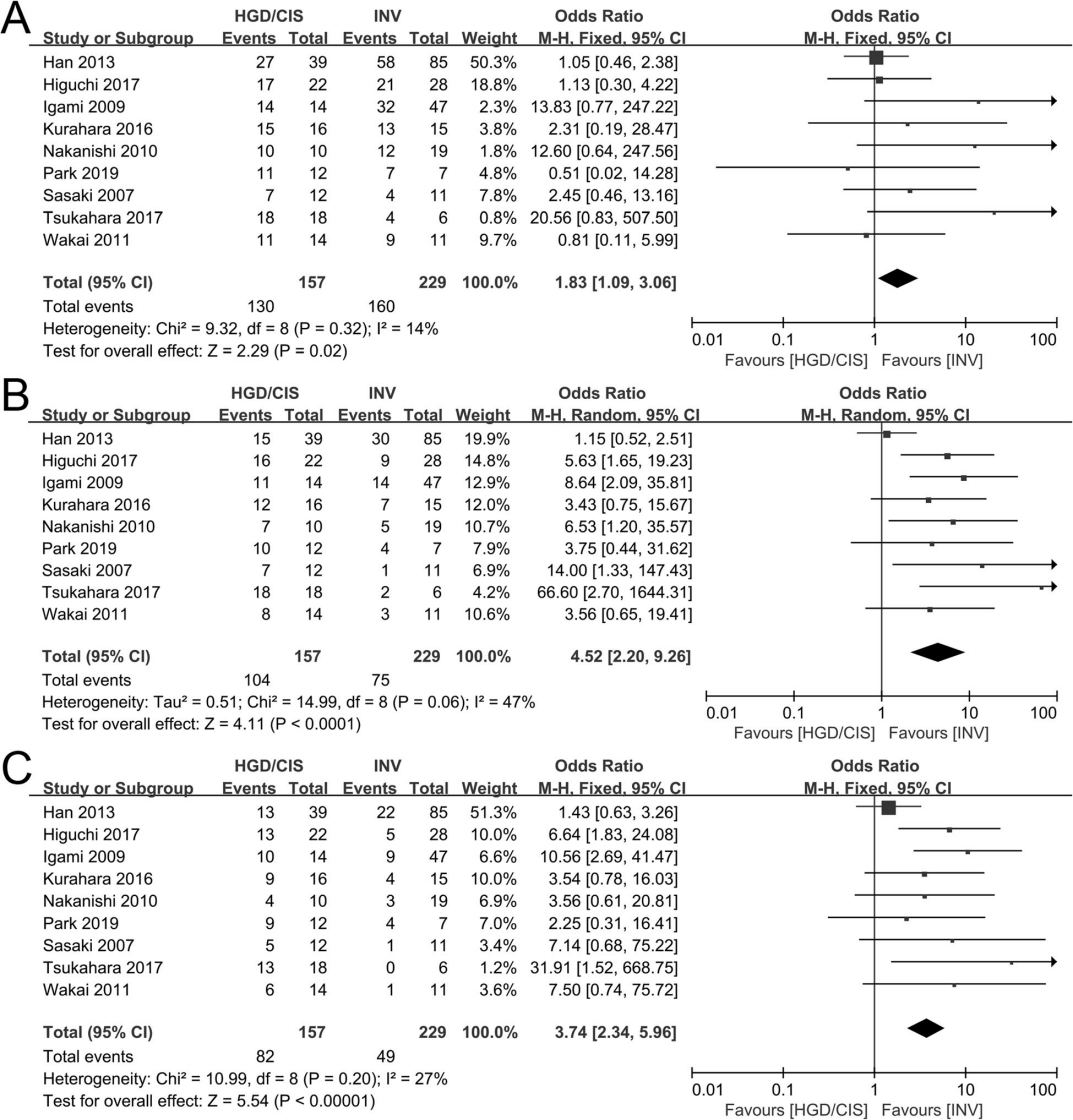
Forest plot of the overall survival rates between HGD/CIS group and INV group. **a** 1-year overall survival rate. **b** 2-year overall survival rate. **c** 3-year overall survival rate

The 1-, 2-, and 3-year survival rates comparing between HGD/CIS group and INV group were evaluated in nine included studies [12, 17–19, 30, 32–35]. Significant heterogeneities were not observed in 1-year and 3-year survival rates (*I*^2^ = 14%, *P* = 0.32; *I*^2^ = 27%, *P* = 0.20; respectively), and using a fixed model, the pooled OR for the 1-year and 3-year survival rates between HGD/CIS group and INV group was 1.83 (95% CI 1.09~3.06, *P* = 0.02, Fig. 3a) and 3.74 (95% CI 2.34~5.96, *P* < 0.0001, Fig. 3c), respectively. A significant heterogeneity was shown in 2-year survival rate (*I*^2^ = 47%, *P* = 0.06), and using a random model, the pooled OR was 4.52 (95% CI 2.20~9.26, *P* < 0.0001, Fig. 3b).

### Subgroup analysis of extrahepatic cholangiocarcinoma at early stage

The 1-, 2-, and 3-year survival rates of patients with pN0M0 comparing between HGD/CIS group and R0 group were evaluated in four included studies [17–19, 34]. Using a fixed model, the pooled OR for the 1-, 2-, and 3-year survival rates between HGD/CIS group and INV group was 0.54 (95% CI 0.21~1.36, *P* = 0.19, Table 2), 0.75 (95% CI 0.35~1.58, *P* = 0.44, Table 2), and 0.74 (95% CI 0.40~1.37, *P* = 0.34, Table 2), respectively.

**Table 2:** Subgroup analysis of the prognosis of extrahepatic cholangiocarcinoma at early stage

Subgroup	Studies included	Overall survival			
		Participants	Effect model	OR (95% CI)	*P*
pN0M0, HGD/CIS vs. R0					
1 year	4	354	Fixed	0.54 (0.21–1.36)	0.19
2 years	4	354	Fixed	0.75 (0.35–1.58)	0.44
3 years	4	354	Fixed	0.74 (0.40–1.37)	0.34
pN0M0, HGD/CIS vs. INV					
1 year	4	85	Fixed	3.47 (1.09–11.02)	*0.03*
2 years	4	85	Fixed	9.12 (2.98–27.93)	*< 0.001*
3 years	4	85	Fixed	9.17 (2.95–28.55)	*< 0.001*

*P* value is statistically significant

The 1-, 2-, and 3-year survival rates of patients with pN0M0 comparing between HGD/CIS group and INV group were evaluated in four included studies [17–19, 34]. Using a fixed model, the pooled OR for the 1-, 2-, and 3-year survival rates between HGD/CIS group and INV group was 3.47 (95% CI 1.09~11.02, *P* = 0.03, Table 2), 9.12 (95% CI 2.98~27.93, *P* < 0.001, Table 2), and 9.17 (95% CI 2.95~28.55, *P* < 0.001, Table 2), respectively.

### Secondary endpoints

**Fig. 4:**
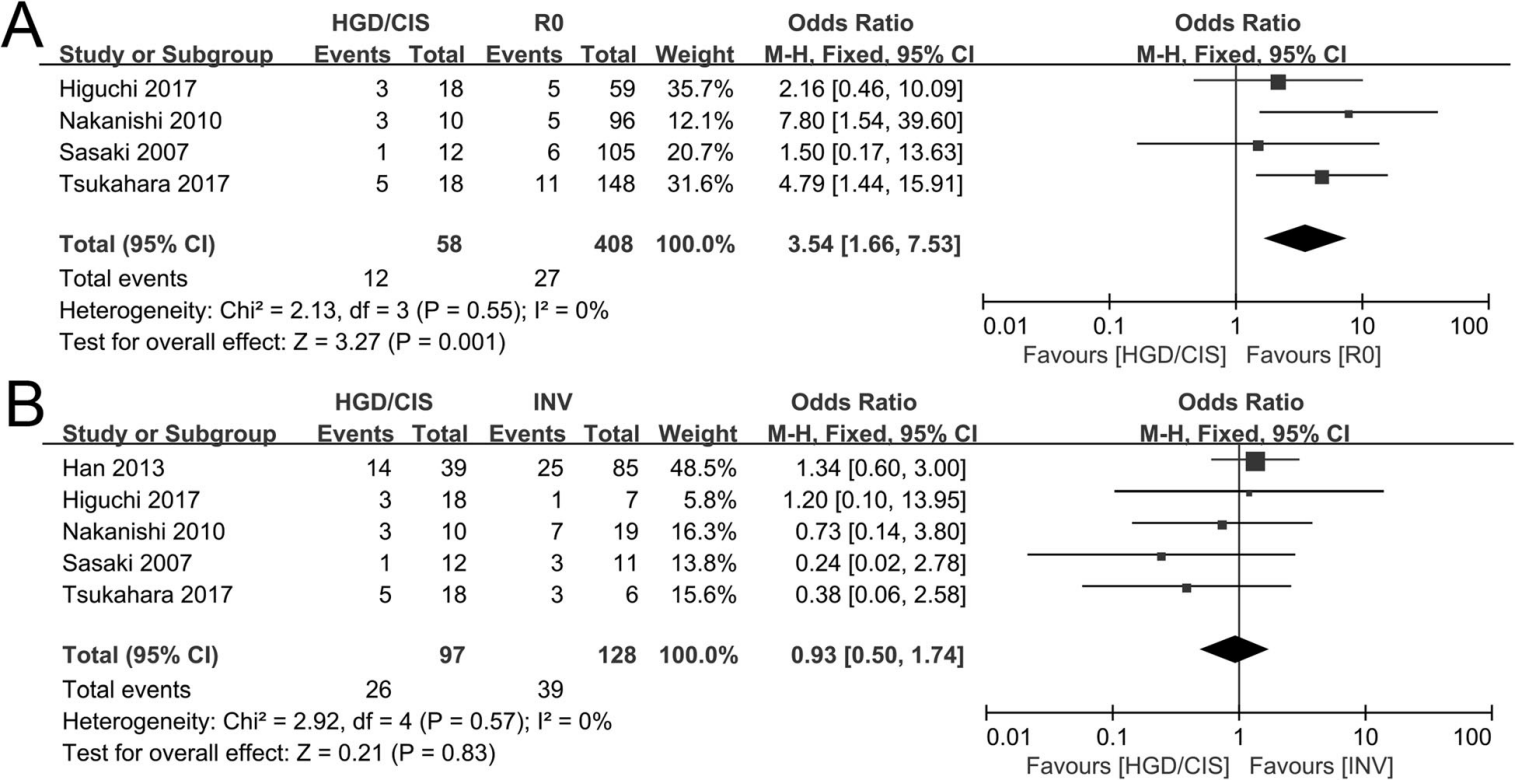
Forest plot of the incidences of recurrence. **a** Between HGD/CIS group and R0 group. **b** Between HGD/CIS group and INV group

The incidences of local recurrence comparing between HGD/CIS group and R0 group were evaluated in four included studies [17, 19, 30, 33]. Significant heterogeneities were not observed (*I*^2^ = 0, *P* = 0.55), and using a fixed model, the pooled OR for the incidence of recurrence between HGD/CIS group and R0 group was 3.54 (95% CI 1.66~7.53, *P* = 0.001, Fig. 4a).

The incidences of local recurrence comparing between HGD/CIS group and INV group were evaluated in five included studies [17, 19, 30, 33, 35]. Significant heterogeneities were not observed (*I*^2^ = 0, *P* = 0.57), and using a fixed model, the pooled OR for the incidence of recurrence between HGD/CIS group and INV group was 0.93 (95% CI 0.50~1.74, *P* = 0.83, Fig. 4b).

### Sensitivity analysis

**Fig. 5:**
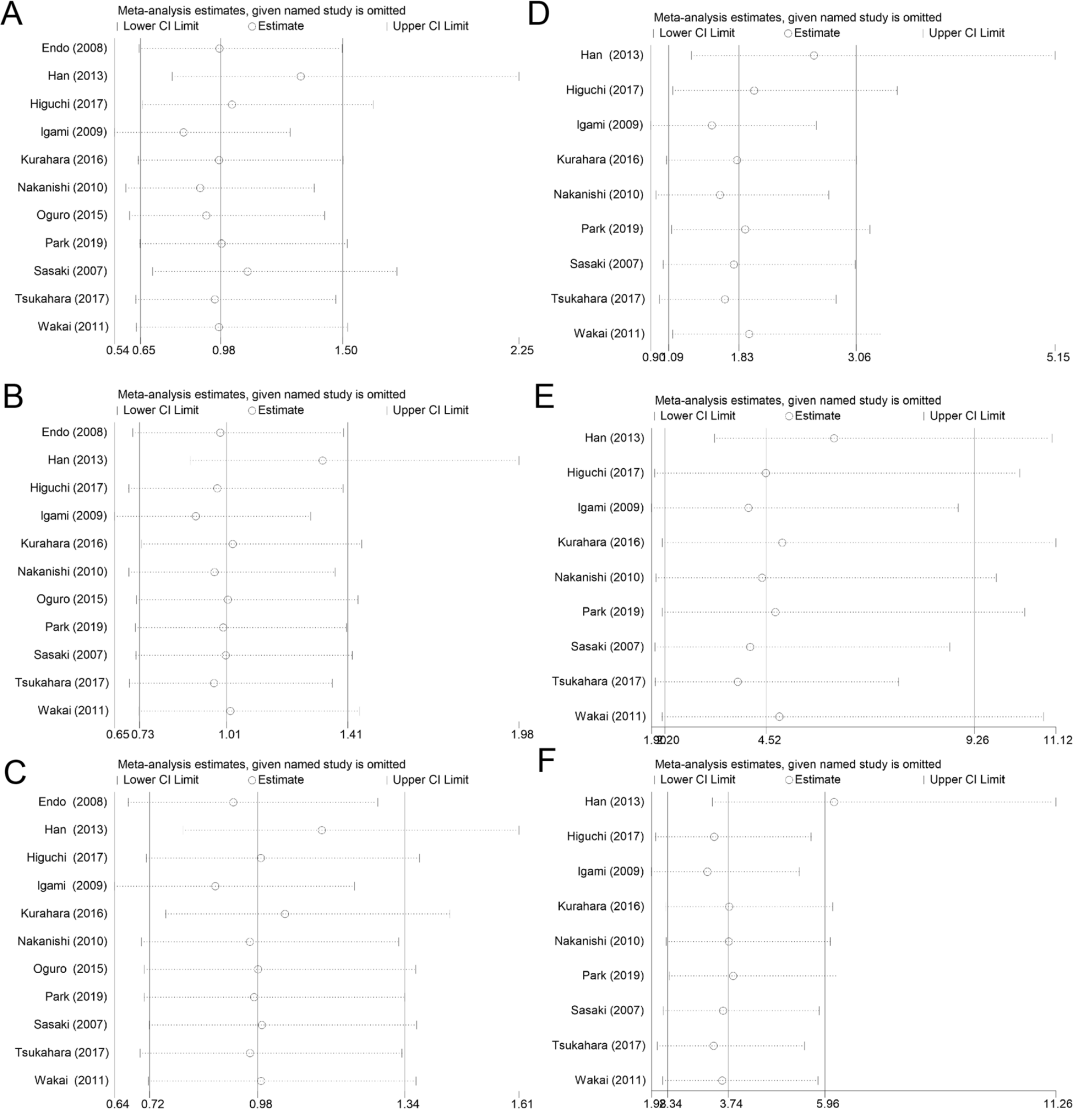
Sensitivity analysis. **a** 1-year overall survival rate between HGD/CIS group and R0 group. **b** 2-year overall survival rate between HGD/CIS group and R0 group. **c** 3-year overall survival rate between HGD/CIS group and R0 group. **d** 1-year overall survival rate between HGD/CIS group and INV group. **e** 2-year overall survival rate between HGD/CIS group and INV group. **f** 3-year overall survival rate between HGD/CIS group and INV group

Sensitivity analysis showed that both the 1-, 2-, and 3-year survival rates comparing between HGD/CIS group and R0 group and the 1-, 2-, and 3-year survival rates comparing between HGD/CIS group and INV group did not change substantially after any study was removed (Fig. 5), which indicated that the results were considerably reliable.

### Meta-analysis of baseline characteristics related to prognosis

Baseline characteristics related to prognosis including the proportion of adjuvant therapy, differentiation, venous invasion, perineural invasion, pT3/4, and pN1/2 were assessed by meta-analysis, and no significant differences were observed both between HGD/CIS group and R0 group and between HGD/CIS group and INV group (Table 3), which indicated that our results were reliable.

**Table 3:** Baseline characteristics related to prognosis

Outcome	Studies	Participants	Effect model	OR (95% CI)
HGD/CIS vs. R0				
Adjuvant therapy	3	377	Fixed	1.55 (0.75–3.20)
Differentiation (moderate/poor)	5	660	Fixed	0.62 (0.36–1.06)
Venous invasion	3	389	Fixed	1.87 (0.83–4.21)
Perineural invasion	4	575	Fixed	1.00 (0.52–1.91)
pT 3/4*	4	494	Fixed	0.57 (0.29–1.10)
pN 1/2*	3	388	Fixed	0.52 (0.24–1.15)
HGD/CIS vs. INV				
Adjuvant therapy	4	203	Fixed	0.79 (0.42–1.47)
Differentiation (moderate/poor)	5	126	Fixed	0.50 (0.23–1.08)
Venous invasion	3	76	Random	0.30 (0.02–4.07)
Perineural invasion	4	95	Fixed	0.44 (0.15–1.33)
pT 3/4*	4	102	Fixed	0.44 (0.19–1.02)
pN 1/2*	3	73	Fixed	0.62 (0.22–1.76)

*According to the 8th edition American Joint Committee on Cancer (AJCC) staging guidelines

### Publication bias analysis

The publication bias analysis was conducted both the 1-, 2, and 3-year survival rates comparing between HGD/CIS group and R0 group and the 1-, 2-, and 3-year survival rates comparing between HGD/CIS group and INV group, and results showed that significant publication bias was not observed in the Begg’s test (Pr > |*z*| = 0.062, 0.087, 0.161, and Pr > |*z*| = 0.175, 0.175, 0.466; respectively). But, in the egger’s test, significant publication bias was observed in the 1- and 2-year survival rates comparing between HGD/CIS group and R0 group and the 2- and 3-year survival rates comparing between HGD/CIS group and INV group (Pr > |*z*| = 0.002, 0.002, and Pr > |*z*| = 0.004, 0.044; respectively), and no significant publication bias was observed in the 3-year survival rates comparing between HGD/CIS group and R0 group and the 1-year survival rates comparing between HGD/CIS group and INV group (Pr > |*z*| = 0.074, and Pr > |*z*| = 0.058, respectively). After “trim and fill” analysis, the pooled HR for the 1- and 2-year survival rates comparing between HGD/CIS group and R0 group and the 2- and 3-year survival rates comparing between HGD/CIS group and INV group was 0.635 (0.363–1.112), 0.750 (0.501–1.124), and 2.368 (1.548–3.622), 2.505 (1.609–3.900); respectively, which indicated that the unpublished studies would not change the results.

## Discussion

Surgical resection is still the only potentially curative treatment for patients with extrahepatic cholangiocarcinoma [7, 8], but the incidence of R1 remains high, which might be the crucial reason for poorer prognosis [10, 11]. However, several recent studies found that HGD/CIS did not affect the prognosis of patients with extrahepatic cholangiocarcinoma [12, 16–18], but it has not yet reached a conclusion. To the best of our knowledge, this was the first meta-analysis evaluating the prognostic value of HGD/CIS for extrahepatic cholangiocarcinoma. Results including 11 studies with 2157 patients showed that 1-, 2-, and 3-year survival rates in the group of HGD/CIS were comparable with that in the group of R0, but were better than in the group of INV. However, the incidence of local recurrence in the HGD/CIS group was comparable with INV group, but was significantly higher than that in the R0 group.

R0 resection is a standard procedure for resectable extrahepatic cholangiocarcinoma [4, 37], but the incidence of R1 remains as high as 10~72% [27, 38]. Reasons are as follows: (1) the extent of cholangiocarcinoma infiltration is hard to diagnose preoperatively [8]; (2) complex anatomy of extrahepatic bile duct often increases the risk of surgery in technique [6]; (3) negative false incidence of intraoperative frozen section remains high [39, 40]. HGD/CIS and INV are both defined as R1 [16, 20, 41], but some argues that they might have different outcomes [12, 17, 18]. As known to all, INV is much more aggressive than HGD/CIS in pathology, and it often takes a long time to progress to INV from HGD/CIS [35]. In this meta-analysis, the incidence of R0, HGD/CIS, and INV was 69.3~90.2%, 3.0~19.5%, and 3.5~18.3%, respectively. Results showed that the pooled ORs of 1-, 2-, and 3-year survival rates between HGD/CIS group and R0 group were 0.98 (*P* = 0.94), 1.01 (*P* = 0.793), and 0.98 (*P* = 0.91), but the pooled OR for the 1-, 2-, and 3-year survival rate between HGD/CIS group and INV group was 1.83 (*P* = 0.02), 4.52 (*P* < 0.0001), and 3.74 (*P* < 0.0001), respectively. In addition, the results did not change sustainably after sensitivity analysis. Hence, HGD/CIS might not affect the prognosis of patients with extrahepatic cholangiocarcinoma, and the conclusion was reliable to some extent.

Tumor stage is usually the key factor for the treatment decision [4, 37]. Some argued that HGD/CIS affected the prognosis of patients with pN0M0 [17–19, 34], partly because the prognosis of patients at early stage might be more likely to be affected by the margin status than those at advanced stage. In this meta-analysis, subgroup analysis of extrahepatic cholangiocarcinoma at early stage was conducted. Results showed that the pooled OR for the 1-, 2-, and 3-year survival rate between HGD/CIS group and R0 group was 0.54 (*P* = 0.19), 0.75 (*P* = 0.44), and 0.74 (*P* = 0.34), respectively, but the 1-, 2-, and 3-year survival rates of patients with pN0M0 comparing between HGD/CIS group and INV group were 3.47 (*P* = 0.03), 9.12 (*P* < 0.001), and 9.17 (*P* < 0.001), respectively, which were coincident with the whole. This indicated that the conclusion that HGD/CIS might not affect the prognosis of patients with extrahepatic cholangiocarcinoma was suitable for the subgroup of early stage, but it should be cautious as for patients at advanced stage.

Recurrence is the Achilles’ heel for hepatobiliary cancers, which is often the just arch-criminal for the poor prognosis [4, 42]. The incidence of 2-year recurrence is reported to be as high as 80% [43, 44], and the R1 is often considered to be one of the important factors for recurrence [9, 11]. In this meta-analysis, local recurrence was taken as the secondary endpoint. Results showed that the pooled OR for the incidence of local recurrence between HGD/CIS group and R0 group was 3.54 (*P* = 0.001), and the pooled OR for the incidence of recurrence between HGD/CIS group and R0 group was 0.93 (*P* = 0.83), which indicated that additional resection should be recommended to achieve R0 if technically possible.

There were several restrictions of this meta-analysis. First, all the included studies were single-center and small sample, indicating an obvious selection bias. Second, 10 of 11 the included studies came from Japan and South Korea [12, 17–19, 30, 32–36], indicating an apparent regional bias because the epidemiology differed between the West and East. Third, the TNM staging systems and surgical procedures were greatly different between hilar cholangiocarcinoma and distal cholangiocarcinoma [4, 6, 8], but most of the included studies had not treated them separately [18, 19, 30, 32–35]. Fourth, the results of the intraoperative frozen section were unreliable because the margin was broken by energy instruments such as CUSA. Fifth, all the included studies were retrospective studies, indicating an apparent recalling bias, although the baseline characteristics were confirmed comparable both between HGD/CIS group and R0 group and between HGD/CIS group and INV group. The last but not the least, publication bias was hard to be avoided, although significant publication bias was not detected after “trim and fill” analysis.

## Conclusion

With the current data, we concluded that HGD/CIS did not affect the prognosis of patients with extrahepatic cholangiocarcinoma regardless of TNM stage, but it increased the risk of local recurrence compared with R0. Hence, additional resection should be recommended if technically possible. In future, to distinguish HGD/CIS from INV is the crucial, and multi-center, larger sample, and prospective randomized trials are warranted to reach a definite conclusion.

## Data Availability

All data generated or analyzed during this study are included in the published articles.

## Abbreviations

HGD: High-grade dysplasia
CIS: Carcinoma in situ
INV: Invasive carcinoma
HCCA: Hilar cholangiocarcinoma
PHC: Perihilar cholangiocarcinoma
OS: Overall survival
OR: Odds ratio
CI: Confidence interval
RCT: Randomized controlled trial
